# Genome topology analysis and transcriptomics of human osteoclasts reveals enhancer–promoter interactions at loci for bone traits and diseases

**DOI:** 10.1093/jbmrpl/ziaf120

**Published:** 2025-07-16

**Authors:** Scott G Wilson, Purdey J Campbell, Dhanya Sooraj, Kassandra Leatherbarrow, Benjamin H Mullin, Suzanne J Brown, Kun Zhu, Shelby Mullin, Bryan K Ward, Jordan Zhang, Jonathon Torchia, Frank Dudbridge, Jiake Xu, Nathan J Pavlos, David Chandler, John P Walsh

**Affiliations:** Department of Endocrinology and Diabetes, Sir Charles Gairdner Hospital, Nedlands, WA, 6009, Australia; School of Biomedical Sciences, University of Western Australia, Nedlands, WA, 6009, Australia; Department of Twin Research and Genetic Epidemiology, King’s College London, London, SE1 7EH, United Kingdom; Department of Endocrinology and Diabetes, Sir Charles Gairdner Hospital, Nedlands, WA, 6009, Australia; Australian Genome Research Facility, Victorian Comprehensive Cancer Centre, Melbourne, Victoria, 3000, Australia; School of Biomedical Sciences, University of Western Australia, Nedlands, WA, 6009, Australia; Department of Endocrinology and Diabetes, Sir Charles Gairdner Hospital, Nedlands, WA, 6009, Australia; School of Biomedical Sciences, University of Western Australia, Nedlands, WA, 6009, Australia; Department of Endocrinology and Diabetes, Sir Charles Gairdner Hospital, Nedlands, WA, 6009, Australia; Department of Endocrinology and Diabetes, Sir Charles Gairdner Hospital, Nedlands, WA, 6009, Australia; Medical School, University of Western Australia, Crawley, WA, 6009, Australia; Department of Endocrinology and Diabetes, Sir Charles Gairdner Hospital, Nedlands, WA, 6009, Australia; School of Biomedical Sciences, University of Western Australia, Nedlands, WA, 6009, Australia; Department of Endocrinology and Diabetes, Sir Charles Gairdner Hospital, Nedlands, WA, 6009, Australia; Harry Perkins Institute of Medical Research, Centre for Medical Research, University of Western Australia, QEII Medical Centre, Nedlands, WA, 6009, Australia; Dovetail Genomics, Scotts Valley, CA, 95066, United States; Dovetail Genomics, Scotts Valley, CA, 95066, United States; Department of Population Health Sciences, University of Leicester, Leicester, LE1 7RH, United Kingdom; School of Biomedical Sciences, University of Western Australia, Nedlands, WA, 6009, Australia; Faculty of Pharmaceutical Sciences, Shenzhen University of Advanced Technology, and Shenzhen Institutes of Advanced Technology, Chinese Academy of Sciences, Shenzhen 518107, China; School of Biomedical Sciences, University of Western Australia, Nedlands, WA, 6009, Australia; Australian Genome Research Facility, Victorian Comprehensive Cancer Centre, Melbourne, Victoria, 3000, Australia; Department of Endocrinology and Diabetes, Sir Charles Gairdner Hospital, Nedlands, WA, 6009, Australia; Medical School, University of Western Australia, Crawley, WA, 6009, Australia

**Keywords:** osteoclast, genome topology, chromatin conformation, Micro-C, chromatin loop, BMD, osteoporosis

## Abstract

Genome-wide association studies (GWAS) relevant to osteoporosis have identified hundreds of loci; however, understanding how these variants influence the phenotype is complicated because most reside in non-coding DNA sequence that serves as transcriptional enhancers and repressors. To advance knowledge on these regulatory elements in osteoclasts (OCs), we performed Micro-C analysis, which informs on the genome topology of these cells and integrated the results with transcriptome and GWAS data to further define loci linked to BMD. Using blood cells isolated from 4 healthy participants aged 31-61 yr, we cultured OC in vitro and generated a Micro-C chromatin conformation capture dataset. We characterized chromatin loops (CLs) in OC from among more than 69 million chromatin interactions identified in the genome. Of the CL identified in OC, >16 000 were unique compared to precursor cells. When sentinel single nucleotide polymorphisms from osteoporosis and bone-related GWAS and those in linkage disequilibrium at *r*^2^ > 0.6 were mapped to CL for OC, 12 588 of these variants were observed within chromatin contact regions. Notable in differential gene ontology enrichment analyses of the topology data for OC and precursors were pathways regulating pluripotency of stem cells, Wnt signaling, nucleotide-binding oligomerization domain (NOD)-like receptor signaling and chemokine signaling. These data, in combination with other 3D genome architecture and epigenetic data (eg, histone modifications and chromatin accessibility), will be useful in modeling to predict genome-wide, which enhancers regulate which genes in OC. This data will therefore also be informative for resolving GWAS hits. In conclusion, we have generated a high-resolution genome topology dataset for human OC and have used this to identify CLs relevant to studies of the genetics of osteoporosis. This data will serve as a powerful resource to inform future functional studies of OC biology.

## Introduction

Osteoporosis is a systemic skeletal disease characterized by low bone mass and microarchitectural deterioration of bone tissue, with a consequent increase in bone fragility and susceptibility to fracture. It is a multifactorial musculoskeletal disorder estimated to affect more than 150 million people worldwide.^1^ Heritability estimates for BMD, the primary risk factor for osteoporosis, indicate a strong contribution of genetic variation to risk for the disorder (approximately 60%-80%).^2^^,^^3^ Although rare genetic variants are known to play a role in the disorder in some individuals,^4^^,^^5^ common variation appears to explain a significant portion of heritability (~20%^6^). There have been many genome-wide association studies (GWAS) examining various phenotypes relevant to bone health and disease; the most recent GWAS of estimated BMD highlighted more than 500 sentinel genome-wide significant single nucleotide polymorphism (SNP).^6^ Understanding the functional relevance of these loci is complicated because most of the highlighted genetic variants reside in non-coding DNA sequence, much of which is speculated to contain transcription regulation elements such as enhancers and repressors. In addition, many of the identified loci contain numerous genetic variants that all show significant association with the trait. As a consequence of linkage disequilibrium (LD)—correlation between the variants—it is unclear which one is the quantitative trait variant, or whether numerous variants in the region are functional and which genes are controlled. Various approaches offer assistance in progressing functional genomics efforts to characterize these loci, including fine mapping (eg, FINEMAP^7^), integration of ENCODE and other regulatory element data (eg, FUMA^8^; RegulomeDB^9^) and colocalization (Coloc^10^), among others.^11^ Osteoblasts are responsible for bone formation by producing new bone matrix, while osteoclasts (OC) break down bone tissue through resorption. The balance between these 2 cell types is crucial for maintaining bone mass, with osteoblast and osteoclast activity contributing to bone growth, maintenance, and repair and excessive osteoclast activity leading to bone loss (eg, osteoporosis). There are numerous omics datasets relevant to osteoblasts that are available to guide progression of functional genomics efforts to characterize these loci,^6^ including chromatin conformation data from Hi-C analysis of MG-63 osteoblast-like cells.^12^ However, to date, there are no publicly available chromatin conformation data for OC. Chromatin conformation data such as Hi-C and Micro-C provide information revealing three dimensional (3D) interactions between regulatory elements (eg, enhancers and promoters) and genes. This is crucial in functional genomics for understanding gene regulation, chromatin organization, and how spatial genome architecture influences cellular function and disease. Hi-C is a widely used chromatin conformation assay that leverages DNA-DNA proximity ligation to map long-range chromatin interactions across the genome, providing insights into the 3D architecture of the genome within the nucleus. In a typical Hi-C experiment, cells are fixed to cross-link spatially adjacent loci, followed by DNA fragmentation and ligation of these proximally located sequences. The resulting chimeric sequences reflect the spatial organization of the genome and the loop anchor or contact regions in the original sample. Analysis of this chromatin conformation data reveals key features of genomic organization,^13^ including A/B compartments, sub-compartments, contact domains, and chromatin loops (CLs).^14^^,^^15^ Therefore, in this study, we sought to perform Micro-C analysis,^16^ a high resolution version of Hi-C,^14^^,^^17^ on human OC to address the need for chromatin conformation data and facilitate efficient targeting of ongoing functional genomics studies relevant to these cells.

## Materials and methods

### Subject recruitment

The protocol for recruitment of participants and laboratory procedures used for the culture of the OC has been described in detail previously.^18^ In brief, for this study, we recruited 2 female and 2 male subjects aged 31-61 yr (mean (SD) = 42.7 (13.1)), for the culture of peripheral blood mononuclear cells (PBMC; OC precursors^19^) and cultured human OC like cells^20^ for studies of chromatin conformation. Subjects were community dwelling individuals, not suffering from medical conditions or using medications likely to affect osteoclastic bone resorption or the ability of their cells to undergo osteoclastogenesis (including bisphosphonates). All subjects had self-reported European ancestry. A sample of whole blood was collected from each patient by venous phlebotomy into EDTA vacutainers and subsequently processed using conventional protocols for OC culture.^18^ The study was approved by the Sir Charles Gairdner and Osborne Park Health Care Group Human Research Ethics Committee and all participants provided written informed consent.

### Isolation of PBMC and osteoclastogenesis

**Figure 1:**
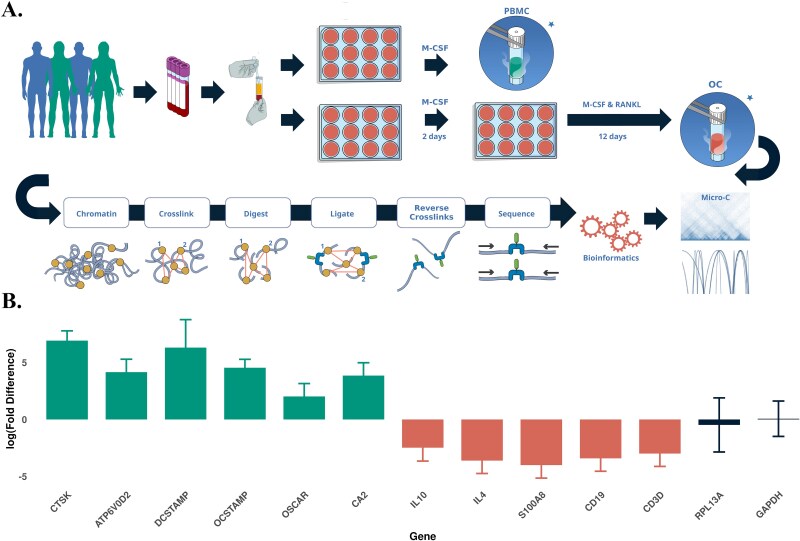
(A) Schematic representation of the research workflow showing the collection of samples from healthy human donors, timeline for the in vitro culture of primary cells, and isolation of PBMCs and osteoclasts (OCs) for RNA-seq (*) and Micro-C analyses. (B) Gene expression data from RNA-seq of OC highlighting differences in genes relevant to OC (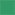) and monocytes (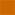), compared to housekeeping genes (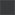); expression of all genes shown (except *GAPDH* and *RPL13A*) are significantly different (*p* < .05) between the OC and PBMC cultures.

Peripheral blood mononuclear cells were isolated from blood tubes by density gradient centrifugation, using protocols established in our laboratory and described previously.^19^ Upon collection, the PBMC were suspended in 1 mL complete α-MEM (minimum essential medium) supplemented with 10 ng/mL macrophage colony stimulating factor (M-CSF), with each sample used to seed 3 wells (triplicates) of a 24-well cell culture plate with 1.5 × 10^6^ cells each. Cultures of PBMC for Micro-C analysis were flash frozen after 2 d. At that time, the medium was replaced in the remaining cultures with α-MEM supplemented with 10 ng/mL M-CSF and 100 ng/mL RANKL, and these cultures for OC were continued for another 12 d while osteoclastogenesis occurred,^18^ and then flash frozen (Figure 1A). Staining of the differentiated cells to confirm production of tartrate resistant acid phosphatase was performed as described previously^18^ (Figure S1), and the cellular gene expression profile was confirmed using RNA-Seq to characterize the OC phenotype of the cultures.

### RNA-Seq

Total RNA was extracted from each set of triplicate cell cultures using the AllPrep DNA/RNA Mini Kit (QIAGEN) according to the manufacturer’s instructions, with on-column DNase digestion for the RNA fraction.^18^ The cell lysate from each set of triplicate cultures was combined into a single aliquot for each sample. RNA integrity numbers (RINs) were assessed for each RNA sample using the Agilent 2100 Bioanalyzer, with all samples recording RIN values ≥9.9 indicating high quality RNA. Quantitation of gene expression was performed by the Australian Genome Research Facility (AGRF) on the RNA samples extracted from the PBMCs and OC cells using 150 bp Paired End RNA-Seq on a NovaSeq X Plus Sequencing System (Illumina). The primary sequence data were generated using the Illumina BCL Convert 4.1.5 pipeline. The cleaned sequence reads were then aligned against the *Homo sapiens* genome (Build version hg38). The STAR aligner (v2.3.5a) was used to map reads to the genomic sequences. The transcripts were assembled with the StringTie tool v2.1.4 (http://ccb.jhu.edu/software/stringtie/) utilizing the reference annotation-based assembly option. The RefSeq annotation containing both coding and non-coding annotations for the Genome Reference Consortium Human Build 38 (hg38) was used as a guide. Gene expression normalization was performed using the edgeR package.^21^ Trimmed Mean of M-(TMM) values normalization was applied to account for compositional differences between samples. To correct for differences in total read count, expression values were converted to counts per million (CPM). Additionally, fragments per kilobase per million values were calculated to facilitate comparison of expression levels between different genes. Analysis of differentially expressed genes (DEG) in PBMC and OC was performed using glmQLFTest in edgeR.

### Micro-C assay

The Micro-C analysis, used to characterize the 3D chromatin architecture of the OCs and the precursor PBMCs, was performed on pellets of the cultured cells using the Dovetail Micro-C Assay (Cantata Bio LLC), which provides ultra-high-resolution topology characterization across the genome. The Micro-C experiments were performed on one million PBMCs and OCs per study subject, that were flash frozen in suspension and used the Micro-C assay kit for mammalian cells as per the manufacturer’s protocol (Figure 1A). Briefly, chromatin was cross-linked using disuccinimidyl glutarate and formaldehyde then digested in situ using an optimized micrococcal nuclease to generate highly uniform fragments. This uniquely captures nucleosome positioning while maintaining an even coverage across the genome, without the sequence bias inherent to restriction enzyme-based Hi-C approaches. Following digestion, the cells were lysed with SDS to extract the chromatin fragments, and these were bound to chromatin capture beads. Next, the chromatin was subjected to proximity ligation, where the bead bound chromatin was end polished, bridge-ligated, and intra-aggregate ligated before crosslink reversal and DNA purification. The purified DNA was then converted into a sequencing library using Illumina-compatible adaptors. Biotin-containing fragments were isolated using streptavidin beads prior to PCR amplification. Libraries were sequenced on a NovaSeq X Plus sequencing system (Illumina) using 150 bp paired-end mode at high depth (300 million read pairs per library) with data generated using the Illumina DRAGEN BCL Convert 07.021.645.4.0.3 pipeline and generated 136.5 (11.6) Gb and 120.0 (10.2) Gb of sequence, respectively. The reads were mapped to the hg38 reference assembly using BWA mem^22^ set to map mates independently and for optimal results with proximity library reads. Pairtools^23^ was used for processing alignments for ligation detection (ie, Micro-C pairs). The pair files were sorted, followed by removal of PCR/optical duplicates. Library QC stats were collected using Micro-C tools^24^ and the library complexity was analyzed using preseq v3.2.0.

### Bioinformatics analysis

We performed analysis of the deep sequence reads and undertook a genome-wide differential analysis of sequence data for the PBMC and the differentiated OC, with CL observed in only one of the cell types referred to as differential loop loci (DLL). Canatata Bio’s proprietary epiPipe software was used for downstream data processing; this pipeline incorporates TopoLink Loop Calling Kit software (Juicer—eigenvector for A/B Compartments; Juicer—arrowhead for Topologically Associating Domains (TADs) and Loop calling, including HiCcompare for normalizing and for differential analysis of Micro-C datasets and MUSTACHE software for multi-scale detection of CLs and Micro-C contact maps). The web-based implementation of PhenoGram (1.2.0)^25^ was used to illustrate genome-wide distribution of CL contact regions in OC that contained one or more SNPs relevant to osteoporosis and bone metabolism traits. The plotgardener (1.8.3) genomic data visualization package in R (4.3.3) was used to graph the data with subsequent gene annotation using GENCODE V46 and GeneHancer^26^ regulatory models (hg38; January 2019 (V2: Corrections to Experiment field)), where relevant. As this was an exploratory analysis where the priority was on sensitivity rather than specificity, CL identification was carried out using a false discovery rate (FDR) of 0.1. We also analyzed publicly available Micro-C data for human embryonic stem cells (H1-ESC; 4DNESEVQ1V15),^27^ for comparison with the OC data. Relevant derivative summary datasets for OC were deposited to the Mendeley Data^28^ and submitted to the Musculoskeletal Knowledge Portal^29^ (https://msk.hugeamp.org/).

### Mapping of candidate osteoporosis GWAS SNPs to CL contact regions

We queried the NHGRI-EBI Catalog of Published GWAS,^30^ using the UCSC Genome Table Browser, for SNPs reported as associated with traits relevant to bone mass, architecture, size and fracture (Table S1) at *p* < 1 × 10^−5^. Those variants were annotated using FUMA^8^ to highlight all potential quantitative trait nucleotides (ie, candidate SNPs), including all independent association signals and SNPs in LD with those at *r*^2^ > 0.6. We then used SNPnexus^31^ to ascertain the position of variants on hg38 and BEDTools^32^ to map those variants to the CL contact regions for OCs. Since it has been noted that different fine-mapping algorithms can provide different sets of fine-mapped variants,^33^ we chose not to restrict the data, but instead examined both the higher (sentinel) and lower (SNPs in LD with sentinel variants at *r*^2^ > 0.6) confidence variants in these studies rather than risk overlooking an important regulatory variant; we therefore report results for all these variants. Publicly available Hi-C data for osteoblast-like cells (MG-63; GSM4417605) was analyzed to determine how many bone GWAS SNPs were located within CL contacts in those cells, providing a comparison with the OC data. We also used the incidence of GWAS SNPs for neuroticism,^30^ a trait unlikely to be related to osteoclast biology, as a comparator for the incidence of bone GWAS SNPs within OC CL contacts.

### Analysis of DEG, DLL, and SNP clusters

To determine the fraction of DEGs near a cluster of candidate SNPs, we clustered the SNPs using BedTools by grouping those within 5000 bp of one another. Clusters with fewer than 10 SNPs were excluded, and the total clustered region size was calculated based on the distance between the first and last SNP. The overlap between these SNP clusters and DEGs was then assessed for all candidate SNPs and only sentinel SNPs. We then used these data in combination with the DLLs to investigate differential regulation between the 2 cell types and help identify which chromatin architecture changes might be driving changes in gene expression. The average distance between a promoter and functionally validated enhancers in the human genome varies widely, with proximal enhancers typically being within 1-2 kb upstream or downstream of the promoter. In contrast distal enhancers, which regulate gene expression over long genomic distances, sometimes acting across introns, intergenic regions, and other genes, are typically from 10 kb to 1 Mb from the target gene transcription start site (median distance is estimated to be around 100-200 kb). Therefore, we examined a range of distances (50, 200, and 1000 kb) for concordance between DLLs and DEGs. We used ChromHMM chromatin state annotations that are available for human CD14+ monocytes, an ontogenetically related cell type, as proxies to illustrate the potential enhancer landscape within CL contacts in the PBMC and OC.

### Differential gene ontology (GO) enrichment analysis of the topology data

Gene ontology (GO) enrichment analysis was used to identify GO terms that were significantly over-represented in the set of CL for the PBMC and differentiated OC. This approach can provide insights into the biological processes, cellular components, and molecular functions that are highlighted by the CL data and can reveal differences between the cell types. The R package clusterProfiler was used for the enrichment analysis of GO terms based on the graph structure of the GO. First, genes were assigned to loops using the “annotatePeak” function of the ChIPseeker R package using Ensembl v86 gene IDs. ChIPseeker annotates the gene associated with the closest promoter (±1 kb TSS) and assigns this gene to each loop. Next, the “compareCluster” function from clusterProfiler was used to perform GO enrichment analysis. The hypergeometric test was used to determine whether any GO terms were significantly over-represented in the OC gene list compared to the PBMC background list. Results were corrected for multiple hypothesis testing with *p*_adjusted_ ≤ .05 considered significant.

## Results

In these studies, we sought to enhance understanding of regulatory elements in OC by analyzing the genome topology of primary cells from 4 healthy donors and integrating the findings with transcriptome and GWAS data to generate high-quality omics data, with the aim of refining loci associated with BMD. RNA-Seq data confirmed the osteoclastic character of those cells and comparative gene expression difference with the PBMC, including high levels of *CTSK*, *ATP6V0D2*, *DCSTAMP*, *OCSTAMP*, *OSCAR*, and *CA2* compared to the PBMC (Figure 1B, Table S2).

Bioinformatics analyses of the Micro-C sequence data characterized a total of 18 045 unique CLs across 3 detection scales (1, 4, and 16 kb) in OC from among ~69 million chromatin interactions that were identified in the genome. Differential analysis of Micro-C data using HiCcompare highlighted 1549 statistically significant differences (*p*_adjusted_) between OC and the precursor cells (the top 30 are shown in Table S3).

**Figure 2:**
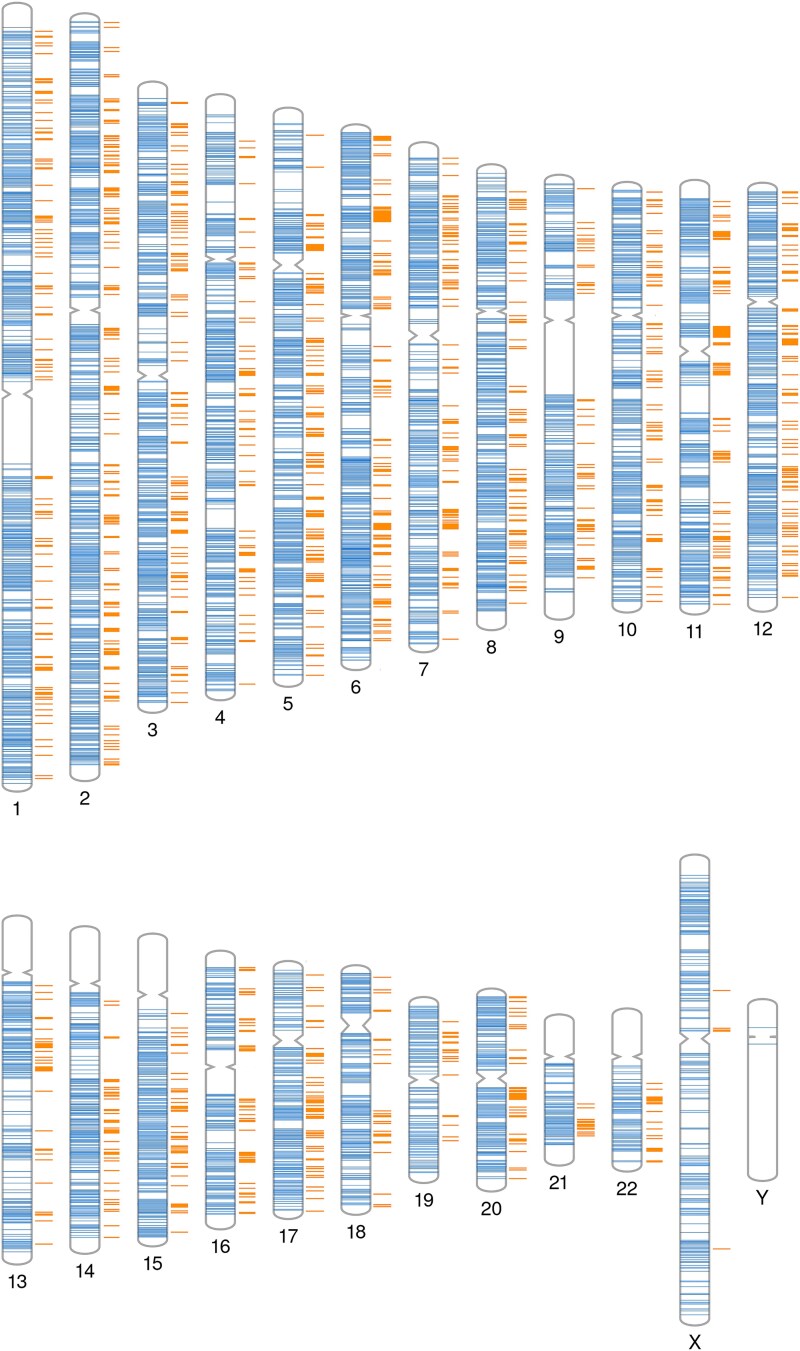
Genomic distribution of all chromatin loops (CL) (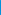) identified in OC and those loop contact regions that contain a GWAS SNP* relevant to bone density and osteoporosis traits (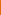). * independent genome-wide associations reported in NHGRI-EBI Catalog of published genome-wide association studies at *p* < 1 × 10^−5^ (sentinel variants) and genetic variants in linkage disequilibrium (LD) at *r*^2^ > 0.6).

Genome topology studies comparing OC to precursor cells highlighted 16 498 loops that were unique to OC. When we compared the Micro-C data from PBMCs and OC with publicly available Micro-C data from H1-ESC cells^27^ and also Hi-C chromatin conformation data from osteoblast-like MG-63 cells,^12^ despite differences in cell type and methodology, we observed broadly similar loop size distributions (Figure S2), supporting the validity of our experimental and analytical approach. Querying the NHGRI-EBI Catalog of Published GWAS for SNPs associated with relevant traits (Table S1) identified 10 810 sentinel variants. Annotation of these variants highlighted 117 835 potential quantitative trait nucleotides (ie, candidate SNPs), encompassing all independent association signals as well as SNPs in LD with them at *r*^2^ > 0.6. We identified 13 564 CL contact regions in OC that contained one or more of those candidate SNPs, relevant to osteoporosis and bone metabolism traits (Figure 2). The GWAS data used for this study includes SNPs that are likely relevant to osteoclast function, as well as to osteoblasts, osteocytes, and other cell functions (eg, pituitary, hypothalamus, and skeletal muscle). Therefore, we did not expect all SNPs in the study set to localize to osteoclast loop contacts. For comparison, we examined data for a trait unrelated to osteoclast biology as a comparator and found that the incidence of neuroticism GWAS SNPs that mapped to OC CL contacts was indeed significantly lower than that for bone GWAS SNPs (*p* > .0001), thereby confirming a level of specificity in the approach used.

**Figure 3:**
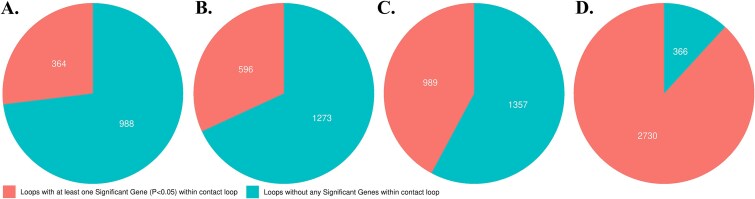
Venn diagrams illustrating the relative proportions of differential loop loci (DLL) in PBMC and OC that have a chromatin contact within (A) a DEG or within (B) 50 kb, (C) 200 kb, and (D) 1000 kb of a DEG.

While, we observed 12 588 candidate SNPs that were within an OC CL contact, we saw only 8100 of the bone GWAS SNPs within CL contacts in the Hi-C data from the osteoblast-like cells (MG-63). We also assessed genes in the vicinity of the OC DLLs to see how many of these were DEGs (Figure 3, Figure S4). Next, we examined the number of SNPs within DLL contacts and found that there were 621 unique candidate SNPs within 172 unique DLL chromatin contacts. Next, we examined clusters of SNPs; examining all candidate SNPs, 2060 clusters were identified, of which 590 unique clusters overlapped with 385 unique DGE. In contrast, when considering just the sentinel GWAS SNPs, 55 clusters were identified, of which 19 unique clusters overlapped with 22 unique DGE. We then evaluated the number of individual candidate SNPs within DEGs, and also those in proximity to DEGs (±50, 200, and 1000 kb). We detected 28 812 unique candidate SNPs in 1900 unique DEGs. In addition, we saw 51 857 unique SNPs within ±50 kb of 3500 unique DEGs. We detected 77 610 unique candidate SNPs within ±200 kb of 5489 unique DEGs, and we detected 112 360 unique candidate SNPs within ±1000 kb of 7991 unique DEGs. Finally, we examined how many DEGs were within or near DLL chromatin contacts. We observed that 364 of 1352 DEGs were within a DLL chromatin contact, 596 of 1869 DEGs were within ±50 kb, 989 of 2346 DEGs were within ±200 kb, and 2730 of 3096 DEGs were within ±1000 kb of a DLL contact (Figure 3).

We were interested to determine the relevance of the OC Micro-C data to the enhancer landscape. As there were no ChromHMM chromatin state annotations available for human OC, we used data from CD14+ monocytes, an ontogenetically related cell type, to illustrate the potential enhancer landscape within CL contacts in both OCs and their PBMC precursors (Figure S3). While these annotations are illustrative rather than definitive, it is notable that over 65% of CL contacts overlapped a ChromHMM-defined enhancer.

**Figure 4:**
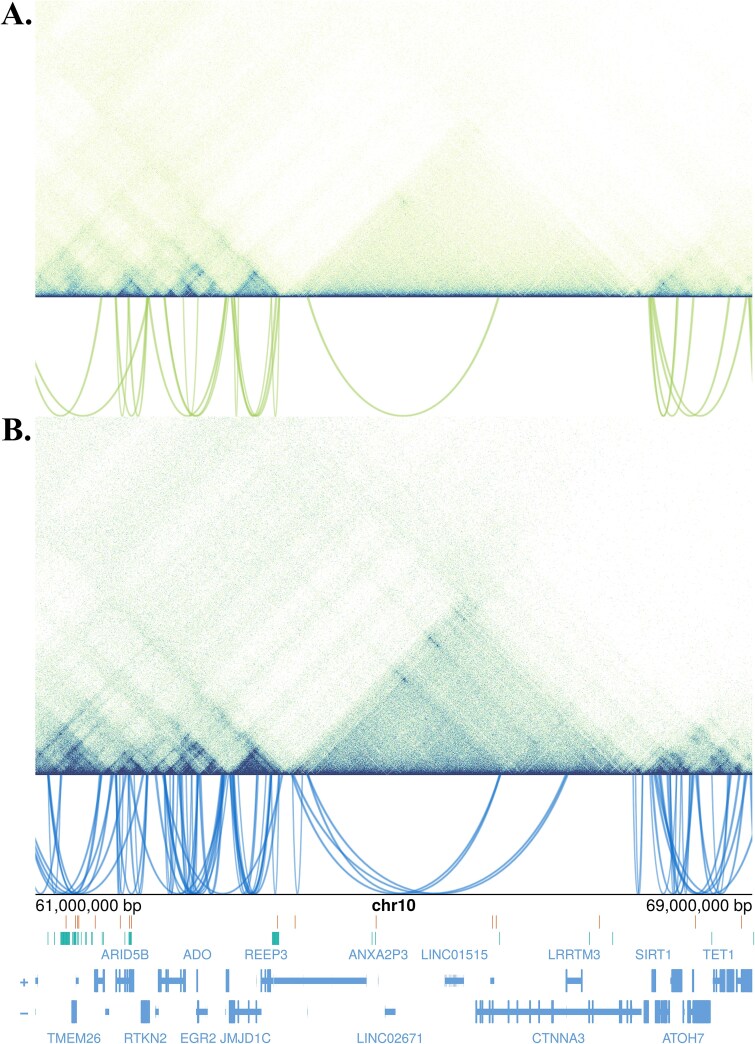
Micro-C data (topographical associated domains and chromatin interaction loops) within a region at chromosome 10q21.2-22.1, generated in (A) human PBMC (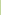) and (B) OC (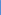). Image shows the sentinel bone GWAS SNPs (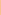) and those in LD (*r*^2^ > 0.6) (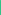) and the genes located on sense (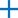) and antisense strand (-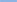) of the DNA.

Among loci in which we observed striking differences in the chromatin topology of the PBMC vs OC, was a region within chromosome 10q21.2-22.1 (61-69 Mb), including the AT-Rich Interaction Domain 5B (*ARID5B*) gene (Figure 4). This locus was recently highlighted in a CRISPRi screen targeted at the genetic etiology of BMD.^34^ This region harbors 16 independent sentinel association signals for bone traits^6^^,^^35–37^ (222 putative candidate regulatory SNPs in total, including those in LD *r*^2^ > 0.6) and contains 66 CL observed in OC vs only 24 in PBMC (*p* < .0001; Figure 4). Only 5 of the loops identified are seen in the two cell types, although 43 of the contact regions are the same in both that is one end of the loop has the same contact, while the other end goes elsewhere. Notably, 39 candidate SNPs for bone traits are mapped within OC loop contacts in this genomic region (Table S4).

**Figure 5:**
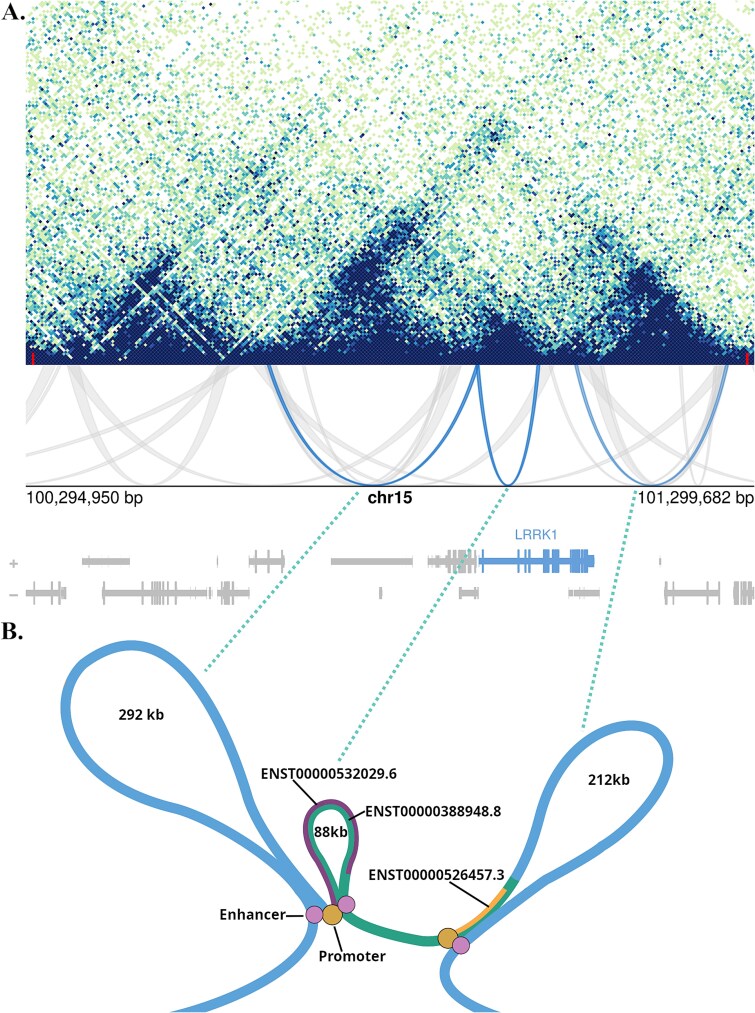
(A) Micro-C data (topographical associated domains (TADs) and chromatin loops (CL) (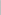) within a region at chromosome 15q26.3 generated in OC. Image shows the genes located on sense (+) and antisense strand (−) of the DNA and TAD domain boundaries at chr15:100305000 and 101290001(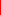); CL showing relevance to *LRRK1* are highlighted (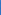). (B) Illustration of the chromatin loops (CL) in association with promoters (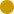) and putative enhancers (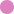) in OC at the *LRRK1* locus at 15q26.3. Note the multiple transcripts reported for *LRRK1* (eg, Gencode transcript: ENST00000388948.8 encoding Q38SD2-1, ENST00000532029.6 encoding Q38SD2-2; ENST00000526457.3 encoding H0YEW1). From left to right enhancer (distal enhancer-like signature—EH38E1793263), promoter (EPDnew promoters LRRK1_1 and LRRK1_3; EH38E1793516/prom, EH38E1793517/prom), enhancer (distal enhancer-like signature—EH38E1793643, EH38E1793644), promoter (EPDnew promoters LRRK1_4), enhancer (putative/no accession No.; contains reported transcription factor binding sites of relevance to OC include ARID2, ESR1, JUN, RELA/RELB, ZNF687).

Data for the CL in OC surrounding the leucine-rich repeat kinase 1 (*LRRK1*) gene at 15q26.3 is informative in illustrating the genome elements that potentially underlie the looping in these cells and shows the interaction of distal enhancers and promoters for 3 reported transcripts of the gene (Figure 5; Gencode Transcript: ENST00000388948.8 encoding Q38SD2-1, ENST00000532029.6 encoding Q38SD2-2; ENST00000526457.3H0YEW1)). *LRRK1* is known to play a role in OC function; the disruption of which results in disease phenotypes in humans (osteopetrosis and osteosclerotic metaphyseal dysplasia)^38^ and mice lacking the gene show severe osteopetrosis, increased bone mineralization, and decreased bone resorption.^39^

**Figure 6:**
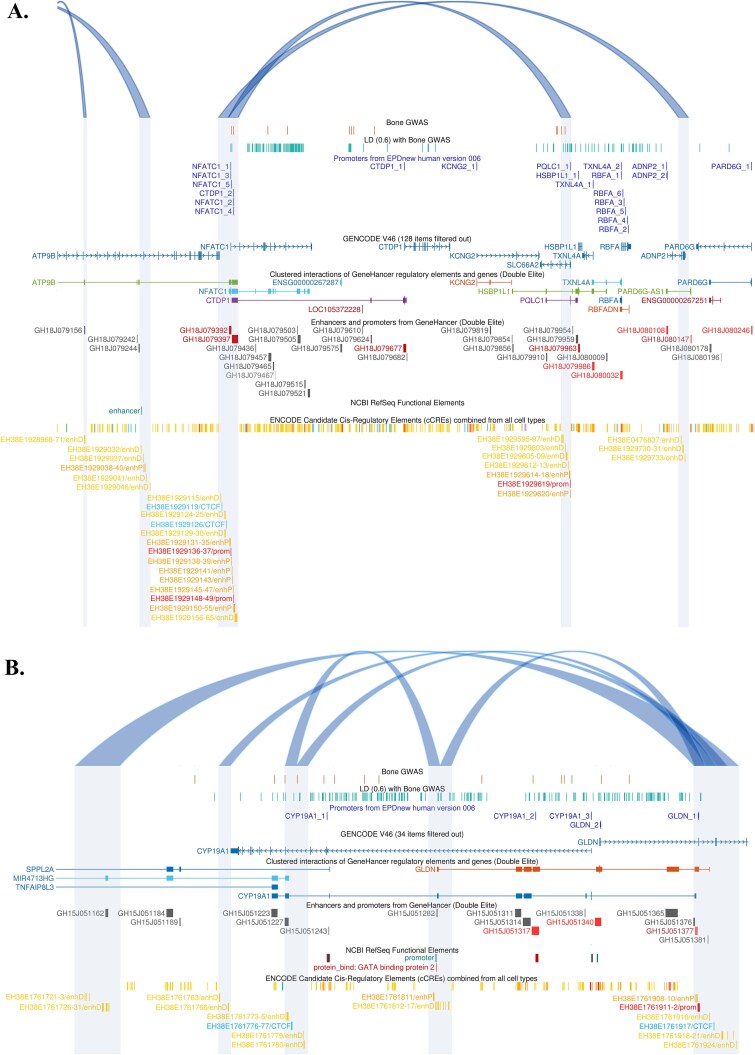
OC chromatin contact data in the region of (A) the *NFATC1* gene at chromosome 18q23 and (B) the *CYP19A1* gene at chromosome 15q21.2. Image shows CL (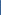) observed in OC in relation to reported sentinel GWAS signals relevant to bone traits (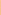), with genetic variants in linkage disequilibrium (LD) at *r*^2^ > 0.6 (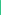), promoters (from EPDnew human version 006), protein coding genes (GENCODE V46) and regulatory elements and gene interactions from GeneHancer (GH Reg Elems (DE) promoters (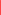) and enhancers (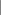)) and ENCODE candidate *cis* regulatory elements (promoters (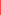) and enhancers (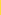)). Shaded regions show the contact regions of the loops.

Another region of substantial interest is within 18q23, the location of the Nuclear Factor of Activated T Cells 1 (*NFATC1*) gene. This locus harbors 203 candidate variants that have been reported as potentially relevant to bone. There are 6 loop contact regions observed in OC compared to only 3 contacts observed in PBMC (*p* = .3270; Figure S5); only 1 contact region is shared at this location between the 2 cell types. Twelve candidate SNPs for osteoporosis and bone traits map within the OC loop contacts in that region (Table S5, Figure 6A).

Among other genomic regions highlighted by GWAS, 15q21.2 (50.5-52.05 Mb) is noteworthy. It contains the Cytochrome P450 Family 19 Subfamily A Member 1 (*CYP19A1*) gene and harbors a total of 244 variants (sentinel GWAS SNPs and those in LD) relevant to bone traits. This genomic sequence has 22 CL observed in OC vs 7 in PBMC (*p* = .0083; Figure S6). Specifically in relation to *CYP19A1*, the localized region of ~250 kb (Figure 6B) has 5 CL observed in OC, vs 1 in PBMC; no CL was shared between the OC and PBMC, but the 2 contact regions were observed in the both cell types. Fourteen variants for bone traits map within the OC loop contacts in that region (Table S6).

Next, to provide further systematic insight into the biological processes, cellular components, and molecular functions that are highlighted by the CL data and highlight differences between the cell types, we undertook GO enrichment analysis (Table S7). Noteworthy in this differential GO enrichment analysis for OC and precursors were pathways regulating pluripotency of stem cells, Wnt signaling, NOD-like receptor signaling, and chemokine signaling (Table S7).

## Discussion

We identified thousands of CL in OC using the Micro-C technique, and this is consistent with the number of loops reported for other cell types such as murine embryonic stem cells,^16^ human embryonic stem cells, and foreskin fibroblasts.^40^ This ultra-deep CL Micro-C mapping has revealed a large number of previously unreported chromosomal features with greatly enhanced signal-to-noise ratios compared to what is purportedly achievable with conventional Hi-C. While chromatin contact data for human osteoblast-like cells, such as Hi-C data from MG-63 cells,^12^ already exist, our osteoclast dataset is unique. The DNA looping interactions that we have characterized are expected to support further research in OC biology, genetic studies of metabolic bone disease, and susceptibility to osteoporosis. Importantly, the vast majority of the CL that were highlighted in OC were not evident from analysis of the precursor PBMC. This observation might be expected based on data from other cell types reported in the literature and confirms that many CL interactions are unique to OC and not discernible through analysis of other cell types which might be considered as more easily accessible proxies. We identified 12 588 bone GWAS SNPs located within CL contacts in OC, compared to 8100 SNPs within CL contacts in osteoblast-like cells (MG-63). However, this difference does not necessarily indicate that a greater proportion of SNPs are functionally relevant in OC. Rather, it likely reflects the higher resolution of Micro-C used to map chromatin interactions in OC, in contrast to the Hi-C method used for OB, which has lower resolution; the higher SNP count in OC likely reflects better loop detection, not necessarily more biological relevance.

A driving force for characterizing genome topology and chromatin contacts in OC is to enhance understanding of regulatory elements and processes relevant to bone resorption and thereby advance knowledge on disease etiology and risk. To this end, we provided examples from 10q21.2 to 10q22.1, 15q21.2, and 18q23, because these regions contain genes of importance in bone metabolism and have been highlighted by GWAS. Within the 10q21.2-22.1 region, there are many GWAS associations reported for bone traits and 2 genes of known relevance to bone. This includes Sirtuin 1 (*SIRT1*), the murine homolog of which has been shown to be a genetic determinant of bone mass in mice, acting in OC through control of NF-κB and in osteoblasts through regulation of cell differentiation,^41^ and *ARID5B*, repression of which has recently been shown to affect osteoblastic phenotypes.^34^ The Micro-C results show the intricate complexity of DNA looping in OC for the region.

The *NFATC1* region is noteworthy because this gene is known to play a crucial role in transcriptional regulation during OC differentiation and activation.^42^^,^^43^ It is perhaps surprising then, that the regulatory complexity of genome topology at this locus appears low in the PBMC, and is similar in the mature cells. It is interesting to see that various putative regulatory elements fall within the observed OC loops (Figure 6A). These include putative promoters (EDPnew ver. 006 NFTAC1_1-5) and 34 ENCODE Candidate Cis-Regulatory Elements (cCREs) in the OC CL chr18:79392000-79 408 000, with 18 other cCREs observed in the other end of the loop at chr18:79936000-79 952 000. Overall, this suggesting the possible interaction of a number of proximal and distal enhancers and promoter elements relevant to *NFTAC1* transcription.

The *CYP19A1* locus was interesting to us because the protein product of this gene is the key enzyme involved in the production of estrogen. This gene encodes the aromatase enzyme, which catalyzes the aromatization of androgens to estrogens, and so presents strongly as having a role in bone, but many would assume that the main action is via the osteoblast and osteocyte. There is also an established relationship between estrogen deficiency and osteoporosis^44^^,^^45^ and aromatase inhibitors, used for therapy in patients with hormone receptor-positive breast carcinoma, are known to increase osteoporosis risk.^46^ Estrogen deficiency is associated with a shift in the balance of bone resorption and bone formation, and although there are multiple possible contributors, estrogen effects on decreasing osteoblast apoptosis, oxidative stress, and osteoblast NF-kB activity appear to be key mediators of the ability of estrogen to maintain bone formation.^47^ The activation of bone remodeling, thought to be mediated via the osteocyte is also understood to be inhibited by estrogen.^47^ Nevertheless, estrogen is also thought to inhibit bone resorption by direct actions on OC,^47^ and we have previously shown eQTLs for *CYP19A1* in OC (eg, rs11636403).^18^^,^^20^ Of note is the OC CL at chr15:51152000-51 168 000 (Figure 6B), which contains a GeneHancer regulatory element (GH15J051162—proposed to be relevant to *MIR4713HG*) and 9 cCREs with distal enhancer-like signatures. That other end of the CL contacts at chr15:51376000-51 392 000, which contains a promoter for *GLDN* (GLDN_1; supported by data from EPDnew human version 006; Genehancer GH15J051377; ENCODE EH38E1761911-2), thus providing another example of a putative distal enhancer–promoter interaction. This OC loop contact at chr15:51376000-51 392 000, also contains GeneHancer regulatory element GH15J051376 (which is likely relevant to *CYP19A1* and *GLDN*) and GH15J051381 (relevant to *GLDN*), plus 3 elements with proximal enhancer-like signatures (EH38E1761908-10) and 6 elements with distal enhancer-like signatures (EH38E1761916, EH38E1761918-21, and EH38E1761924). This fairly simple looping circuitry appears to show promoter–enhancer control relevant to *GLDN* with coordinated control of *CYP19A1* and *MIR4713HG*. Recent studies demonstrated colocalization between eBMD GWAS and OC eQTL^20^ signals within the *CYP19A1*/*GLDN* locus, implying a role in these cells. Figure 6B also shows an OC loop that has contact at chr15:51228001-51232000 and contains a GeneHancer regulatory element (GH15J051227; thought to be relevant to *CYP19A1* and *MIR4713HG*), and 3 ENCODE cCREs with distal enhancer-like signatures (EH38E1761773-5). At the other end of that loop, at chr15:51280001-51 284 000, there is a putative promoter element (*CYP19A1* promoter I.7 (LOC110386949); thus providing another example of a possible distal enhancer–promoter loop interaction. This OC loop contact at chr15:51280001-51284000 also contains a GeneHancer regulatory element (GH15J051282), purportedly relevant to *CYP19A1* and *GLND*, plus ENCODE cCREs (EH38E1761811 with a proximal enhancer-like signature and EH38E1761812-4 with distal enhancer-like signatures), overall illustrating the coordinated control of multiple genes in OC. Consistent with these known effects of estrogen on bone, we found increased numbers of CL in OCs in this region compared to the precursor cells, suggesting that mature OC are under complex control and are likely responsive to the various transcription factors that interact with the regulatory elements highlighted by this Micro-C data in a coordinated way.

Strengths of this study include the high resolution (ie, Micro-C) analysis at which the analysis was performed, the age and sex composition of the study subjects, and availability of complementary eQTL,^18^^,^^20^ meQTL, and RNAseq^19^ data. In this report, we have focused on analysis and reporting of DNA looping in *cis* for OC from healthy individuals; however, looping in *trans* is well known in some cell types, particularly for the small, gene-rich chromosomes.^13^ Our deep sequence data provides a potential path into studies of chromosomal looping in *trans* in OC, which is yet to be undertaken. In multinucleated OC, formalin cross-linking in the Micro-C protocol stabilizes spatially proximate DNA regions within each nucleus, allowing for analysis of intra-nuclear chromatin interactions. Despite multiple nuclei in each cell, individual nuclei remain distinct, each with its own nuclear envelope and chromatin territory. Therefore, inter-nuclear looping is unlikely to occur or be captured by standard Micro-C protocols, as chromatin from different nuclei does not come into contact in a way that would produce detectable ligation events. Limitations of this work include the relatively modest number of individuals studied (there were 4 participants in this study and while 2 or 3 biological replicates is common in the field, the number could be increased to improved CL discovery), and the constraint of the study to subjects of Northern European ancestry. As this was an exploratory analysis, we used a relatively liberal FDR and so a replication study is needed to confirm the results at a stricter level of significance. Future research in this realm should examine larger numbers of study subjects, diverse ancestries, well-powered sex-specific analyses, and consider disease-specific states, analyzing not only *cis* interactions, but also *trans* interactions.

## Conclusion

We have generated a unique high-resolution Micro-C genome topology dataset for human OC, capable of aiding in the identification of enhancer–promoter interactions at loci for bone traits and diseases. We have used this Micro-C DNA looping information to highlight a number of putative genetic regulatory variants relevant to osteoporosis and bone disease. Approximately 11% of putative regulatory variants identified through various bone trait related GWAS fall within the CL of OCs that we have identified in this study; a substantial proportion of the total given the expectation that many of these signals seen to date would be expected to be relevant to other cells in bone (ie, osteoblast, osteocyte) and to other cell types in the body (mesenchymal and hematopoietic stem cells, cells of the hypothalamic-pituitary-gonadal axis, etc.). We also examined the level of concordance between the location of candidate SNPs, DLLs, and DEGs. Information on fraction of SNPs that overlap with DEGs may provide some insight into how many GWAS SNPs are relevant to genes that are osteoclast related. This data could aid in identifying osteoclast-related SNPs which could be segregated in future from SNPs specific for genes expressed in osteoblasts.

A number of the genes highlighted in this study are thought or known to have a role in bone biology, including *LRRK1*,^38^  *ARID5B*,^34^  *SIRT1*,^41^  *NFATC1*,^48^ and *CYP19A1*.^18^^,^^49^ However, further research is needed to achieve more complete knowledge of the transcriptional control of these genes in OC. In particular, genome-wide data on epigenetic markers, including histone modifications, is needed to provide critical insights into the transcriptional control of genes in OC by identifying active and repressed chromatin states, enhancer–promoter interactions, and regulatory elements. This OC Micro-C dataset should contribute to that goal; these data will complement the growing body of evidence from many sources embodied in the Musckuloskeletal Knowledge Portal.^29^ The results from this study should aid in advancing the use of models for resolving GWAS loci relevant to osteoporosis and bone traits and disease in which effector gene(s) or regulatory mechanisms are currently unclear, and so will help target future translational studies.

## Supplementary Material

Supplementary_Material_for_Genome_topology_analysis_ASBMR-24100821_R4_ziaf120

## Data Availability

The raw sequence underlying the findings reported in this article cannot be shared publicly due to human research ethics constraints and privacy issues relating to the individuals that participated in the study. The CL summary data for OC that support the findings of this study are openly available in Mendeley Data.
