# Supplementary material for: Genome topology analysis and transcriptomics of human osteoclasts reveals enhancer–promoter interactions at loci for bone traits and diseases
Source: JBMR Plus. 2025 Jul 16;9(10):ziaf120. doi: 10.1093/jbmrpl/ziaf120 (PMC12409414; doi:10.1093/jbmrpl/ziaf120)
Supplement: Supplementary_Material_for_Genome_topology_analysis_ASBMR-24100821_R4_ziaf120 [file supplementary_material_for_genome_topology_analysis_asbmr-24100821_r4_ziaf120.docx]

**Supplementary Material for:**

**Genome topology analysis and transcriptomics of human osteoclasts reveals enhancer-promoter interactions at loci for bone traits and diseases.**

Scott G Wilson ^1,2,3,#^, Purdey J Campbell ^1^, Dhanya Sooraj ^4^, Kassandra Leatherbarrow^2^, Benjamin H Mullin ^1,2^, Suzanne J Brown ^1^, Kun Zhu ^1,5^, Shelby Mullin ^1,2^, Bryan K Ward ^1,6^, Jordan Zhang ^7^, Jonathon Torchia ^7^, Frank Dudbridge ^8^, Jiake Xu ^2,9^, Nathan J Pavlos ^2^, David Chandler ^4^, John P Walsh ^1,5.^

**Supplementary tables**

**Table S1.** Bone and osteoporosis traits included from NHGRI-EBI Catalog of Published Genome-Wide Association Studies for the identification of associated SNPs.

Trait Count % Cumulative %

Bone mineral density mean 5589 39.51 39.51

Heel bone mineral density 4665 32.98 72.48

Bone mineral density variability 880 6.22 78.7

Femur bone mineral density x serum urate levels interaction 722 5.1 83.81

Total body bone mineral density 253 1.79 85.59

Heel bone mineral density x serum urate levels interaction 168 1.19 86.78

Bone mineral density (spine) 126 0.89 87.67

Bone density Z-score 98 0.69 88.37

Bone density T-score 96 0.68 89.04

Femoral neck bone mineral density 91 0.64 89.69

Bone stiffness index 79 0.56 90.25

Bone mineral density (hip) 79 0.56 90.8

Skull bone mineral density 78 0.55 91.36

Bone mineral density 76 0.54 91.89

Lumbar spine bone mineral density 74 0.52 92.42

Bone mineral density (femoral neck) 64 0.45 92.87

Fracture of forearm 57 0.4 93.27

L1-L4 bone mineral density x serum urate levels interaction 46 0.33 93.6

Hip bone size 43 0.3 93.9

Fractures 36 0.25 94.15

Bone mineral content 36 0.25 94.41

Bone mineral density (hip) and hip bone size 35 0.25 94.66

Heel bone mineral density (MTAG) 34 0.24 94.9

Bisphosphonate-associated atypical femoral fracture 34 0.24 95.14

Total body bone mineral density (MTAG) 33 0.23 95.37

Ultradistal forearm bone mineral density 32 0.23 95.6

Bone ultrasound measurement (broadband ultrasound attenuation) 28 0.2 95.79

Bone mineral density (paediatric, total body less head) 25 0.18 95.97

Pediatric bone mineral content (radius) 23 0.16 96.13

Bone mineral density (total hip) 22 0.16 96.29

Bone ultrasound measurement (velocity of sound) 21 0.15 96.44

Lumbar spine bone mineral density (integral) 20 0.14 96.58

Bone properties (heel) 20 0.14 96.72

Bone mineral density (paediatric, skull) 19 0.13 96.85

Total body bone mineral density (age over 60) 19 0.13 96.99

Bone mineral density (Ward's triangle area) 19 0.13 97.12

Femoral neck bone mineral density and trunk fat mass adj. by TLM 19 0.13 97.26

Total body bone mineral density (age 45-60) 18 0.13 97.38

Pediatric bone mineral density (femoral neck) 18 0.13 97.51

Pediatric areal bone mineral density (radius) 16 0.11 97.62

Pediatric bone mineral content (hip) 15 0.11 97.73

Hip bone mineral density and total body fat mass (bivariate) 15 0.11 97.84

Pediatric bone mineral density (hip) 15 0.11 97.94

Atypical femoral fracture in phosphonate treatment 14 0.1 98.04

Pediatric bone mineral density (spine) 14 0.1 98.14

Pediatric bone mineral content (femoral neck) 13 0.09 98.23

Ulna and radius bone mineral density 13 0.09 98.32

Lumbar spine bone mineral density (trabecular) 12 0.08 98.41

Pediatric bone mineral content (spine) 12 0.08 98.49

Bone mineral density (paediatric, lower limb) 11 0.08 98.57

Bone mineral density (paediatric, upper limb) 11 0.08 98.65

Spine bone size 10 0.07 98.72

Heel bone mineral density T score 10 0.07 98.79

Bone fracture in osteoporosis 9 0.06 98.85

Bone mineral density (spine) and age at menarche 9 0.06 98.92

Bone mineral accretion in asthma (oral corticosteroid dose int.) 9 0.06 98.98

Total body bone mineral density (age 30-45) 8 0.06 99.04

Bone mineral density (hip) and age at menarche 8 0.06 99.1

Total body bone mineral density (age 0-15) 7 0.05 99.14

Bone mineral density (femoral neck) in IBD 7 0.05 99.19

Peak hip bone mineral content 7 0.05 99.24

Fractures (paediatric) 7 0.05 99.29

Heel bone mineral density variance 6 0.04 99.34

Femoral neck bone geometry and menarche (age at onset) 6 0.04 99.38

Hip bone mineral content accrual during adolescent growth spurt 5 0.04 99.41

Hip fracture 5 0.04 99.45

Bone mineral density at left trochanter (model 2) 4 0.03 99.48

Bone mineral density (lumbar spine) in IBD 4 0.03 99.51

Pediatric areal bone mineral density (RSHDSP) 4 0.03 99.53

Body mass index and bone mineral density (pleiotropy) 3 0.02 99.55

Fractures (vertebral) 3 0.02 99.58

Compression fracture 3 0.02 99.6

Bone mineral density at right trochanter (model 2) 3 0.02 99.62

Bone mineral density (trunk) 3 0.02 99.64

Bone mineral density x blood lead int. in current smokers (2df) 3 0.02 99.66

Bone mineral density at lumbar spine (model 1) 3 0.02 99.68

Bone mineral density at right femoral neck (model 2) 2 0.01 99.7

Bone density 2 0.01 99.71

Pediatric areal bone mineral density (SRHDSP) 2 0.01 99.72

Femoral neck bone geometry 2 0.01 99.74

Bone mineral density at left intertrochanter (model 2) 2 0.01 99.75

Forearm bone mineral density 2 0.01 99.77

Pediatric areal bone mineral density (concordant skeletal phenotype) 2 0.01 99.78

Bone erosion in rheumatoid arthritis 2 0.01 99.8

Gynoid bone mass 2 0.01 99.81

Bone mineral density or leg lean mass (pleiotropy) 2 0.01 99.82

Bone mineral density at right femoral neck (model 1) 1 0.01 99.83

Bone mineral density at right trochanter (model 1) 1 0.01 99.84

Bone mineral density at right total hip (model 1) 1 0.01 99.84

Bone mineral density at left femoral neck (model 1) 1 0.01 99.85

Bone mineral density at right total hip (model 2) 1 0.01 99.86

Bone mineral density at left trochanter (model 1) 1 0.01 99.87

Bone mineral density at left total hip (model 2) 1 0.01 99.87

Bone mineral density at left total hip (model 1) 1 0.01 99.88

Bone mineral density or trunk lean mass (pleiotropy) 1 0.01 99.89

Bone mineral density at right intertrochanter (model 2) 1 0.01 99.89

ICD10 S22: Fracture of rib(s), sternum and thoracic spine 1 0.01 99.9

Bone mineral density T score in postmenopausal osteoporosis 1 0.01 99.91

Android bone mass 1 0.01 99.92

Bone mineral density x blood lead int. in current smokers (1df) 1 0.01 99.92

Bone mineral density (pelvis) 1 0.01 99.93

Bone mineral density at left femoral neck (model 2) 1 0.01 99.94

Bone mineral density at left intertrochanter (model 1) 1 0.01 99.94

Bone mineral density (wrist) 1 0.01 99.95

Spine bone mineral density and alcohol drinking 1 0.01 99.96

Trunk bone mineral content 1 0.01 99.96

Bone mineral density at lumbar spine (model 2) 1 0.01 99.97

Calcaneal bone ultrasound measurement (speed of sound) 1 0.01 99.98

First fracture in long-term childhood cancer survivors (time to event) 1 0.01 99.99

Arm bone mineral content 1 0.01 99.99

Bone mineral density at right intertrochanter (model 1) 1 0.01 100

Abbreviations: adjusted (adj.), trunk lean mass (TLM), interaction (int.), inflammatory bowel disease (IBD), radius vs spine & hip discordant skeletal phenotype (RSHDSP), spine vs radius & hip discordant skeletal phenotype (SRHDSP), MTAG (multi-trait analysis of GWAS).

**Table S2.** The top 50 differentially expressed genes in osteoclasts (OC) verses peripheral blood mononuclear cells (PBMC) determined by RNAseq.

| Gene | logFC | F | p-value | FDR |
| --- | --- | --- | --- | --- |
| *TACC2* | 10.00 | 1102.82 | 1.06E-14 | 4.19E-10 |
| *SLC30A3* | 10.46 | 891.86 | 1.65E-13 | 3.27E-09 |
| *SULT1C2* | 11.10 | 616.57 | 2.60E-13 | 3.43E-09 |
| *DUSP13* | 10.56 | 647.16 | 4.18E-13 | 4.13E-09 |
| *LOC105369812* | 9.79 | 608.10 | 6.31E-13 | 4.99E-09 |
| *COBL* | 9.70 | 792.59 | 9.70E-13 | 6.39E-09 |
| *AFAP1L1* | 11.99 | 1485.96 | 1.58E-12 | 8.92E-09 |
| *VIT* | 10.10 | 654.57 | 3.14E-12 | 1.55E-08 |
| *SERPINB2* | -13.44 | 383.14 | 5.19E-12 | 2.28E-08 |
| *GUCY1A2* | 6.95 | 420.77 | 8.50E-12 | 3.36E-08 |
| *VWCE* | 6.75 | 1287.19 | 1.97E-11 | 6.55E-08 |
| *HES2* | 12.00 | 320.50 | 1.99E-11 | 6.55E-08 |
| *SEZ6L2* | 8.62 | 1181.99 | 3.05E-11 | 9.28E-08 |
| *KDR* | 8.88 | 335.11 | 3.69E-11 | 1.01E-07 |
| *CAPN11* | 7.24 | 540.90 | 4.09E-11 | 1.01E-07 |
| *GAPT* | -8.37 | 483.35 | 4.40E-11 | 1.01E-07 |
| *RNF128* | 11.13 | 731.29 | 4.56E-11 | 1.01E-07 |
| *CTDSPL* | 4.27 | 1049.08 | 4.62E-11 | 1.01E-07 |
| *LOC105369764* | 7.83 | 318.07 | 5.63E-11 | 1.17E-07 |
| *DENND2B* | 5.73 | 996.67 | 5.91E-11 | 1.17E-07 |
| *LOC105372801* | -8.66 | 267.68 | 6.65E-11 | 1.22E-07 |
| *SPATA18* | 8.57 | 882.43 | 6.77E-11 | 1.22E-07 |
| *RGS20* | 10.61 | 382.50 | 8.64E-11 | 1.48E-07 |
| *TSPAN12* | 6.97 | 295.11 | 9.34E-11 | 1.54E-07 |
| *LINC02245* | 8.10 | 255.08 | 9.93E-11 | 1.57E-07 |
| *XCR1* | -8.32 | 874.78 | 1.04E-10 | 1.59E-07 |
| *SHC4* | 8.15 | 322.58 | 1.15E-10 | 1.69E-07 |
| *NMRK2* | 11.39 | 280.00 | 1.21E-10 | 1.70E-07 |
| *NANOS1* | 7.77 | 771.99 | 1.56E-10 | 2.12E-07 |
| *LOC102723370* | 8.91 | 242.36 | 1.82E-10 | 2.33E-07 |
| *LOC101928143* | 5.58 | 783.98 | 1.83E-10 | 2.33E-07 |
| *EFR3B* | 8.30 | 778.00 | 2.22E-10 | 2.74E-07 |
| *SPARC* | 6.52 | 738.62 | 2.41E-10 | 2.89E-07 |
| *ITGA3* | 8.05 | 725.59 | 2.62E-10 | 3.00E-07 |
| *ARFGEF3* | 8.44 | 649.39 | 2.65E-10 | 3.00E-07 |
| *LOC112267968* | 6.05 | 691.11 | 3.56E-10 | 3.91E-07 |
| *TRIM54* | 10.24 | 530.71 | 3.96E-10 | 4.14E-07 |
| *DIRAS2* | 11.08 | 360.54 | 3.98E-10 | 4.14E-07 |
| *CAMK2B* | 8.98 | 233.95 | 4.40E-10 | 4.41E-07 |
| *LGALSL* | 6.21 | 650.79 | 4.46E-10 | 4.41E-07 |
| *BEAN1* | 9.98 | 228.54 | 4.65E-10 | 4.42E-07 |
| *GAL* | 12.90 | 530.46 | 4.73E-10 | 4.42E-07 |
| *NCCRP1* | -8.17 | 201.74 | 4.81E-10 | 4.42E-07 |
| *NCAPH* | 8.26 | 627.90 | 5.18E-10 | 4.65E-07 |
| *SYNGR1* | 6.39 | 620.45 | 5.48E-10 | 4.81E-07 |
| *TTC28* | 4.83 | 611.74 | 5.86E-10 | 5.01E-07 |
| *FCER1A* | -10.10 | 202.82 | 6.04E-10 | 5.01E-07 |
| *ZNF608* | -5.19 | 222.69 | 6.08E-10 | 5.01E-07 |
| *SLC28A3* | 8.01 | 588.68 | 7.02E-10 | 5.66E-07 |
| *CPE* | 8.89 | 519.60 | 8.01E-10 | 6.33E-07 |

**Table S3.** The top 30 loci from differential analysis of Micro-C (chromatin loop) data for

PBMC vs OC.

| Chr | Start 1 | End 1 |  | Start 2 | End 2 | Z | p-value | p_adjusted_ |
| --- | --- | --- | --- | --- | --- | --- | --- | --- |
| chr9 | 17528000 | 17536000 |  | 17800000 | 17808000 | 5.593 | 2.2E-8 | 2.4E-4 |
| chr8 | 111440000 | 111448000 |  | 111784000 | 111792000 | 5.456 | 4.9E-8 | 6.2E-4 |
| chr7 | 147240000 | 147248000 |  | 148080000 | 148088000 | 5.331 | 9.8E-8 | 4.5E-4 |
| chr6 | 122864000 | 122872000 |  | 123312000 | 123320000 | 5.318 | 1.1E-7 | 1.3E-3 |
| chr2 | 212144000 | 212152000 |  | 212560000 | 212568000 | 5.315 | 1.1E-7 | 2.0E-3 |
| chr4 | 165488000 | 165496000 |  | 166088000 | 166096000 | 5.268 | 1.4E-7 | 1.5E-3 |
| chr3 | 192184000 | 192192000 |  | 192448000 | 192456000 | 5.251 | 1.5E-7 | 3.1E-3 |
| chr1 | 74768000 | 74776000 |  | 75200000 | 75208000 | 5.216 | 1.8E-7 | 2.7E-3 |
| chr15 | 34616000 | 34624000 |  | 34912000 | 34920000 | 5.189 | 2.1E-7 | 1.4E-3 |
| chr4 | 36408000 | 36416000 |  | 37384000 | 37392000 | 5.149 | 2.6E-7 | 1.5E-3 |
| chr8 | 92064000 | 92072000 |  | 93360000 | 93368000 | 5.144 | 2.7E-7 | 5.9E-4 |
| chr2 | 187136000 | 187144000 |  | 187448000 | 187456000 | 5.105 | 3.3E-7 | 7.4E-3 |
| chr13 | 86864000 | 86872000 |  | 87272000 | 87280000 | 5.070 | 4.0E-7 | 3.1E-3 |
| chr7 | 94448000 | 94456000 |  | 95200000 | 95208000 | 5.043 | 4.6E-7 | 2.5E-3 |
| chr12 | 114656000 | 114664000 |  | 115744000 | 115752000 | 5.040 | 4.7E-7 | 1.1E-3 |
| chr3 | 192192000 | 192200000 |  | 192424000 | 192432000 | 5.021 | 5.2E-7 | 1.1E-2 |
| chr8 | 110048000 | 110056000 |  | 110824000 | 110832000 | 5.017 | 5.3E-7 | 2.9E-3 |
| chr16 | 19904000 | 19912000 |  | 20296000 | 20304000 | 5.006 | 5.6E-7 | 2.5E-3 |
| chr8 | 108888000 | 108896000 |  | 109312000 | 109320000 | 4.968 | 6.8E-7 | 7.5E-3 |
| chr6 | 121432000 | 121440000 |  | 122024000 | 122032000 | 4.967 | 6.8E-7 | 6.5E-3 |
| chr5 | 87776000 | 87784000 |  | 88720000 | 88728000 | 4.911 | 9.1E-7 | 4.6E-3 |
| chr2 | 15640000 | 15648000 |  | 16480000 | 16488000 | 4.909 | 9.2E-7 | 7.4E-3 |
| chr5 | 128216000 | 128224000 |  | 128504000 | 128512000 | 4.873 | 1.1E-6 | 1.9E-2 |
| chr9 | 70640000 | 70648000 |  | 71248000 | 71256000 | 4.854 | 1.2E-6 | 6.5E-3 |
| chr1 | 74760000 | 74768000 |  | 75192000 | 75200000 | 4.836 | 1.3E-6 | 1.0E-2 |
| chr14 | 63792000 | 63800000 |  | 64288000 | 64296000 | 4.833 | 1.4E-6 | 6.7E-3 |
| chr8 | 84632000 | 84640000 |  | 85048000 | 85056000 | 4.832 | 1.4E-6 | 1.5E-2 |
| chr8 | 84648000 | 84656000 |  | 85048000 | 85056000 | 4.830 | 1.4E-6 | 1.6E-2 |
| chr1 | 237840000 | 237848000 |  | 238944000 | 238952000 | 4.813 | 1.5E-6 | 5.3E-3 |
| chr15 | 56248000 | 56256000 |  | 56416000 | 56424000 | 4.793 | 1.6E-6 | 1.4E-2 |

Data represent the start region and end region of chromatin loops that are statistically significantly different in PBMC and OC. Statistical differences were determined with HiCcompare. Z - for each pairwise interaction point (i.e. a specific region in the contact maps), the software calculates a z-score to quantify how different the interaction frequency is between the two cell types. The z-score is calculated using the mean and variance of the interaction frequencies across the biological replicates, and therefore standardises the difference between the PBMC and OC relative to the variability observed. A positive z-score represents increased interaction in the OC. p_adjusted_ - the

p-value adjusted for multiple testing.

**Table S4.** Genetic variants for bone traits observed within OC loop contacts in the focus region within 10q21.2-22.1.

| OC loop contact region | rsID | Regulation Rank (score)* |
| --- | --- | --- |

chr10:61136000-61152000 rs112186575 7 (0.184)

chr10:61280000-61296000 rs113046488 7 (0.184)

chr10:61280000-61296000 rs1915455 5 (0.59)

chr10:61280000-61296000 rs3107456 1f (0.553)

chr10:61940000-61944000 rs112779912 1f (0.667)

chr10:62032000-62048000 rs10569497 2a (0.476)

chr10:62032000-62048000 rs10657313 1f (0.554)

chr10:62032000-62048000 rs10821946 2b (0.513)

chr10:62032000-62048000 rs12355313 1f (0.554)

chr10:62032000-62048000 rs12357548 1f (0.554)

chr10:62032000-62048000 rs2393743 1a (0.93)

chr10:62032000-62048000 rs4948496 1f (0.554)

chr10:62032000-62048000 rs56140430 1f (0.554)

chr10:62032000-62048000 rs6479781 1d (0.09)

chr10:62032000-62048000 rs6479782 1b (0.76)

chr10:62048000-62064000 rs10740059 1b (0.958)

chr10:62048000-62064000 rs10761602 4 (0.609)

chr10:62048000-62064000 rs10761603 2b (0.621)

chr10:62048000-62064000 rs10761604 4 (0.609)

chr10:62048000-62064000 rs10821948 4 (0.609)

chr10:62048000-62064000 rs10821949 7 (0.514)

chr10:62048000-62064000 rs10821950 1d (0.108)

chr10:62048000-62064000 rs10821952 2b (0.812)

chr10:62048000-62064000 rs10995019 2b (0.633)

chr10:62048000-62064000 rs10995020 4 (0.609)

chr10:62048000-62064000 rs10995021 4 (0.609)

chr10:62048000-62064000 rs16916931 4 (0.609)

chr10:62048000-62064000 rs3740355 4 (0.609)

chr10:62048000-62064000 rs3832683 2b (1)

chr10:62048000-62064000 rs4948498 1f (0.667)

chr10:62048000-62064000 rs576932207 2b (1)

chr10:62048000-62064000 rs713240 1f (0.554)

chr10:62048000-62064000 rs725529 1f (0.554)

chr10:62048000-62064000 rs7898906 4 (0.609)

chr10:62048000-62064000 rs7902719 1b (1)

chr10:62048000-62064000 rs7902904 1f (0.554)

chr10:62048000-62064000 rs7908159 4 (0.609)

chr10:62048000-62064000 rs7922857 4 (0.705)

chr10:63712000-63728000 rs7068032 7 (0.514)

* RegulomeDB prediction annotation of variants using known functional elements from various cell lineages, providing a rank which details the level of supporting data from 1a (strongest) to 7 (weaker) and a probabilistic score (0-1) calculated from a random forest model indicating the likelihood that the variant may be causal.

**Table S5.** Genetic variants for bone traits observed within OC loop contacts in a locus at 18q23.

| OC loop contact region | rsID | Regulation Rank (score)* |  |
| --- | --- | --- | --- |

chr18:79392000-79408000 rs11276781 1b (0.71)

chr18:79392000-79408000 rs56376587 1b (0.575)

chr18:79392000-79408000 rs71359461 1f (0.667)

chr18:79392000-79408000 rs8096658 1f (0.554)

chr18:79936000-79952000 rs12968195 1f (0.554)

chr18:79936000-79952000 rs12969321 1b (0.95)

chr18:79936000-79952000 rs12970432 1b (0.995)

chr18:79936000-79952000 rs34397434 1f (0.553)

chr18:79936000-79952000 rs34704161 1f (0.554)

chr18:79936000-79952000 rs71367559 1b (0.84)

chr18:79936000-79952000 rs75916065 1b (0.98)

chr18:79936000-79952000 rs8095510 1f (0.554)

* RegulomeDB prediction annotation of variants using known functional elements from various cell lineages, providing a rank which details the level of supporting data from 1a (strongest) to 7 (weaker) and a probabilistic score (0-1) calculated from a random forest model indicating the likelihood that the variant may be causal.

**Table S6.** Genetic variants for bone traits observed within OC loop contacts in locus 15q21.2.

| OC loop contact region | RS ID | Regulation Rank (score)* | | | |
| --- | --- | --- | --- | --- | --- |
| chr15:51228000-51232000 | rs1065778 | | 1f (0.554) |  |  |
| chr15:51228000-51232000 | rs12900487 | | 1f (0.554) |  |  |
| chr15:51232000-51236000 | rs2414095 | | 1f (0.554) |  |  |
| chr15:51232000-51236000 | rs12592697 | | 1f (0.222) |  |  |
| chr15:51232000-51236000 | rs72529508 | | N/A |  |  |
| chr15:51280000-51284000 | rs150972916 | | N/A |  |  |
| chr15:51280000-51284000 | rs35384027 | | 1a (1) |  |  |
| chr15:51284000-51288000 | rs2445752 | | 1f (0.222) |  |  |
| chr15:51284000-51288000 | rs2445753 | | 1f (0.029) |  |  |
| chr15:51284000-51288000 | rs936306 | | 1f (0.667) |  |  |
| chr15:51284000-51288000 | rs936307 | | 1f (0.554) |  |  |
| chr15:51376000-51380000 | rs12443032 | | 1f (0.554) |  |  |
| chr15:51376000-51380000 | rs72729227 | | 1f (0.554) | |  |
| chr15:51456000-51472000 | rs62018113 | | 7 (0.514) | |  |

* RegulomeDB prediction annotation of variants using known functional elements from various cell lineages, providing a rank which details the level of supporting data from 1a (strongest) to 7 (weaker) and a probabilistic score (0-1) calculated from a random forest model indicating the likelihood that the variant may be causal.

**Table S7.** Differential GO enrichment analysis of topology data for OC versus PBMC

highlighting enriched pathways/disease associations/biological entities.

ID Description Gene Ratio p-value p_adjusted_

hsa04010 MAPK signalling pathway 295/8083 6.4E-6 2.2E-3

hsa05132 Salmonella inf. 243/8083 1.3E-4 1.5E-2

hsa04110 Cell cycle 156/8083 1.8E-4 1.5E-2

hsa04148 Efferocytosis 155/8083 2.0E-4 1.5E-2

hsa04014 Ras signalling pathway 232/8083 2.5E-4 1.5E-2

hsa05224 Breast cancer 146/8083 3.5E-4 1.5E-2

hsa04810 Regulation of actin cytoskeleton 225/8083 3.6E-4 1.5E-2

hsa04020 Calcium signalling pathway 248/8083 3.7E-4 1.5E-2

hsa04510 Focal adhesion 199/8083 3.9E-4 1.5E-2

hsa04024 cAMP signalling pathway 221/8083 4.5E-4 1.5E-2

hsa04140 Autophagy - animal 163/8083 7.1E-4 2.2E-2

hsa04080 Neuroactive ligand-receptor interaction 356/8083 9.1E-4 2.4E-2

hsa05170 Human immunodeficiency virus 1 inf. 208/8083 9.2E-4 2.4E-2

hsa04015 Rap1 signalling pathway 206/8083 1.0E-3 2.4E-2

hsa05160 Hepatitis C 156/8083 1.1E-3 2.4E-2

hsa04390 Hippo signalling pathway 155/8083 1.2E-3 2.4E-2

hsa04360 Axon guidance 179/8083 1.2E-3 2.4E-2

hsa04151 PI3K-Akt signalling pathway 348/8083 1.3E-3 2.4E-2

hsa04921 Oxytocin signalling pathway 152/8083 1.4E-3 2.4E-2

hsa04144 Endocytosis 244/8083 1.4E-3 2.4E-2

hsa05226 Gastric cancer 147/8083 1.9E-3 2.8E-2

hsa04310 Wnt signalling pathway 171/8083 1.9E-3 2.8E-2

hsa04540 Gap junction 88/8083 2.0E-3 2.8E-2

hsa05210 Colorectal cancer 86/8083 2.3E-3 2.8E-2

hsa05412 Arrhythmogenic right ventricular card. 86/8083 2.3E-3 2.8E-2

hsa05167 Kaposi sarcoma-ass. Herpes virus inf. 190/8083 2.4E-3 2.8E-2

hsa04530 Tight junction 167/8083 2.4E-3 2.8E-2

hsa04012 ErbB signalling pathway 85/8083 2.5E-3 2.8E-2

hsa04814 Motor proteins 189/8083 2.5E-3 2.8E-2

hsa04550 Signalling reg. pluripotency stem cell 141/8083 2.6E-3 2.8E-2

hsa04062 Chemokine signalling pathway 188/8083 2.7E-3 2.8E-2

hsa05225 Hepatocellular carcinoma 165/8083 2.7E-3 2.8E-2

hsa04670 Leukocyte transendothelial migration 114/8083 2.8E-3 2.8E-2

hsa04936 Alcoholic liver disease 140/8083 2.8E-3 2.8E-2

hsa04371 Apelin signalling pathway 137/8083 3.3E-3 3.2E-2

hsa04621 NOD-like receptor signalling pathway 182/8083 3.6E-3 3.4E-2

hsa01521 EGFR tyrosine kinase inhibitor res. 79/8083 3.8E-3 3.4E-2

hsa05165 Human papillomavirus inf. 320/8083 4.1E-3 3.7E-2

hsa05212 Pancreatic cancer 76/8083 4.7E-3 3.9E-2

hsa05220 Chronic myeloid leukaemia 76/8083 4.7E-3 3.9E-2

hsa05205 Proteoglycans in cancer 198/8083 4.8E-3 4.0E-2

hsa04115 p53 signalling pathway 75/8083 5.0E-3 4.0E-2

hsa04218 Cellular senescence 153/8083 5.1E-3 4.1E-2

hsa04740 Olfactory transduction 422/8083 5.4E-3 4.2E-2

hsa04974 Protein digestion and absorption 102/8083 5.9E-3 4.4E-2

hsa05218 Melanoma 72/8083 6.2E-3 4.5E-2

Abbreviations: associated (ass.), infection (inf.), cardiomyopathy (card.), resistance (res.) regulating (reg.). p_adjusted_ - the p-value adjusted for multiple testing.

**Supplementary figures**


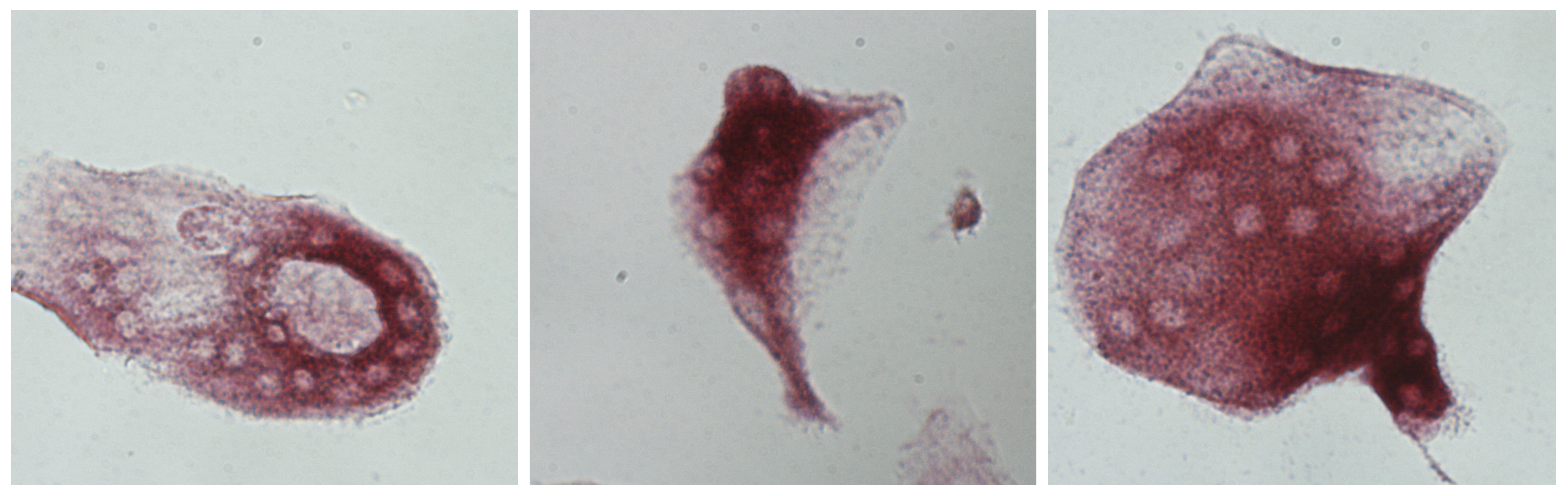
**Figure S1.** Representative images of multinucleated OC used in the Micro-C experiments, stained for ACP5 (TRAP; maroon); imaged using a 40× objective lens.

50 µm


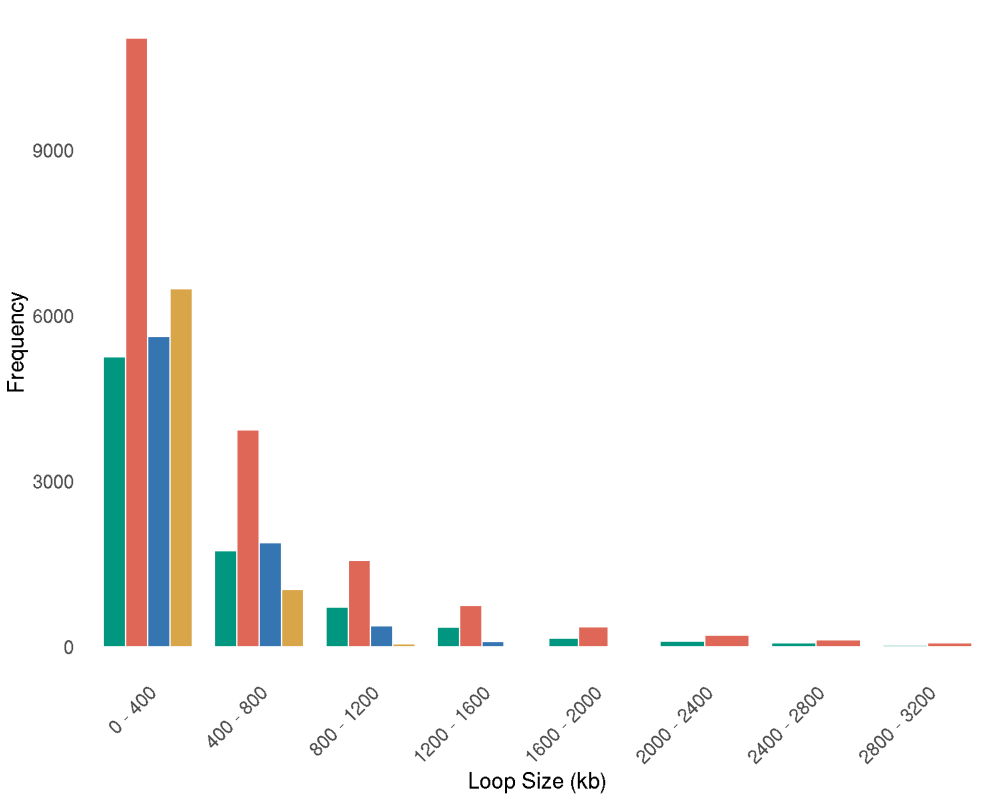


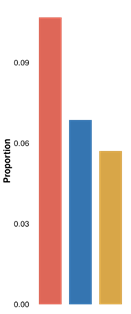


OC OB H1-ESC

A. B.

**Figure S2.** (A) Frequency and size distribution of chromatin loops detected in PBMC (●; Micro-C), OC (●; Micro-C), osteoblast-like cells MG-63 (OB) (● ; Hi-C) and human embryonic stem cells (H1-ESC) (●; Micro-C), (B) Proportion of bone trait and osteoporosis GWAS SNPs located within chromatin loop contacts in OC, OB, and H1-ESC.

A. B.

**
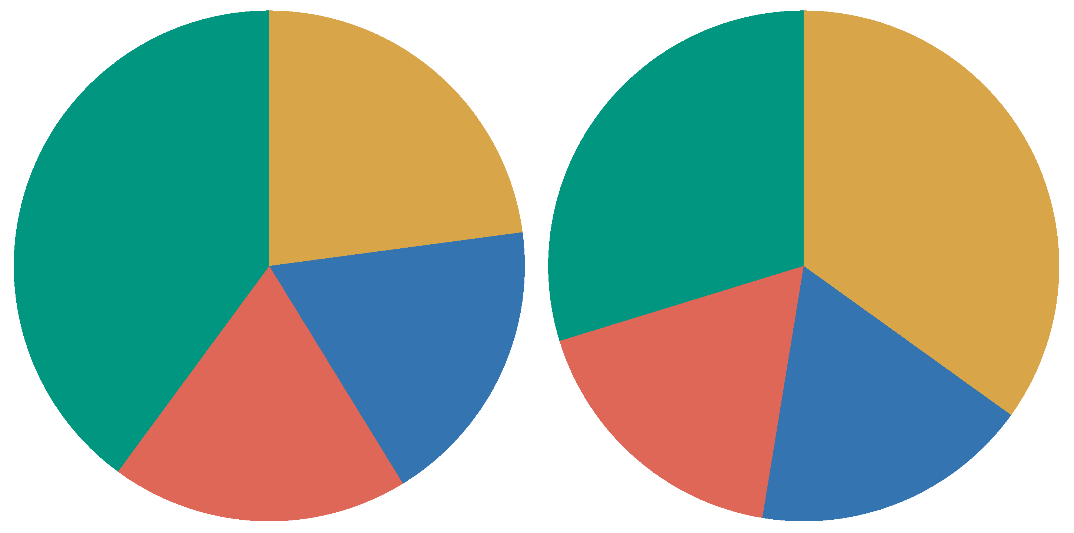
**

**Figure S3.** Relative distribution of ChromHMM-defined enhancers (ENCODE CD14⁺ monocytes) within chromatin loop (CL) contacts in (A) PBMC and (B) OC. Legend: ● no enhancer at either contact; ● enhancer at loop end only; ● enhancer at loop start only; ● enhancer at both loop contacts.


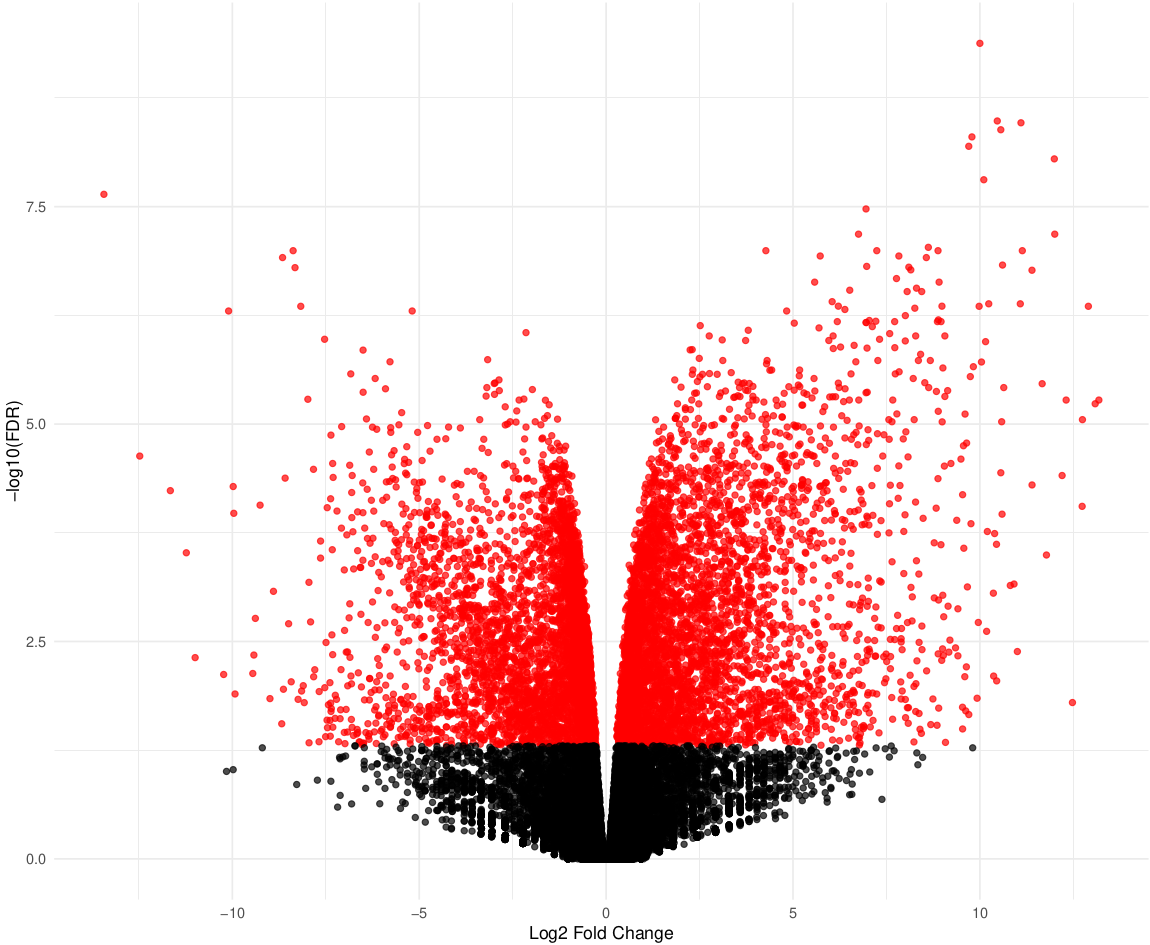


**Figure S4.** Volcano plot showing statistically significant increased or decreased differential expression of genes (DEG) in OC versus PBMC (●).


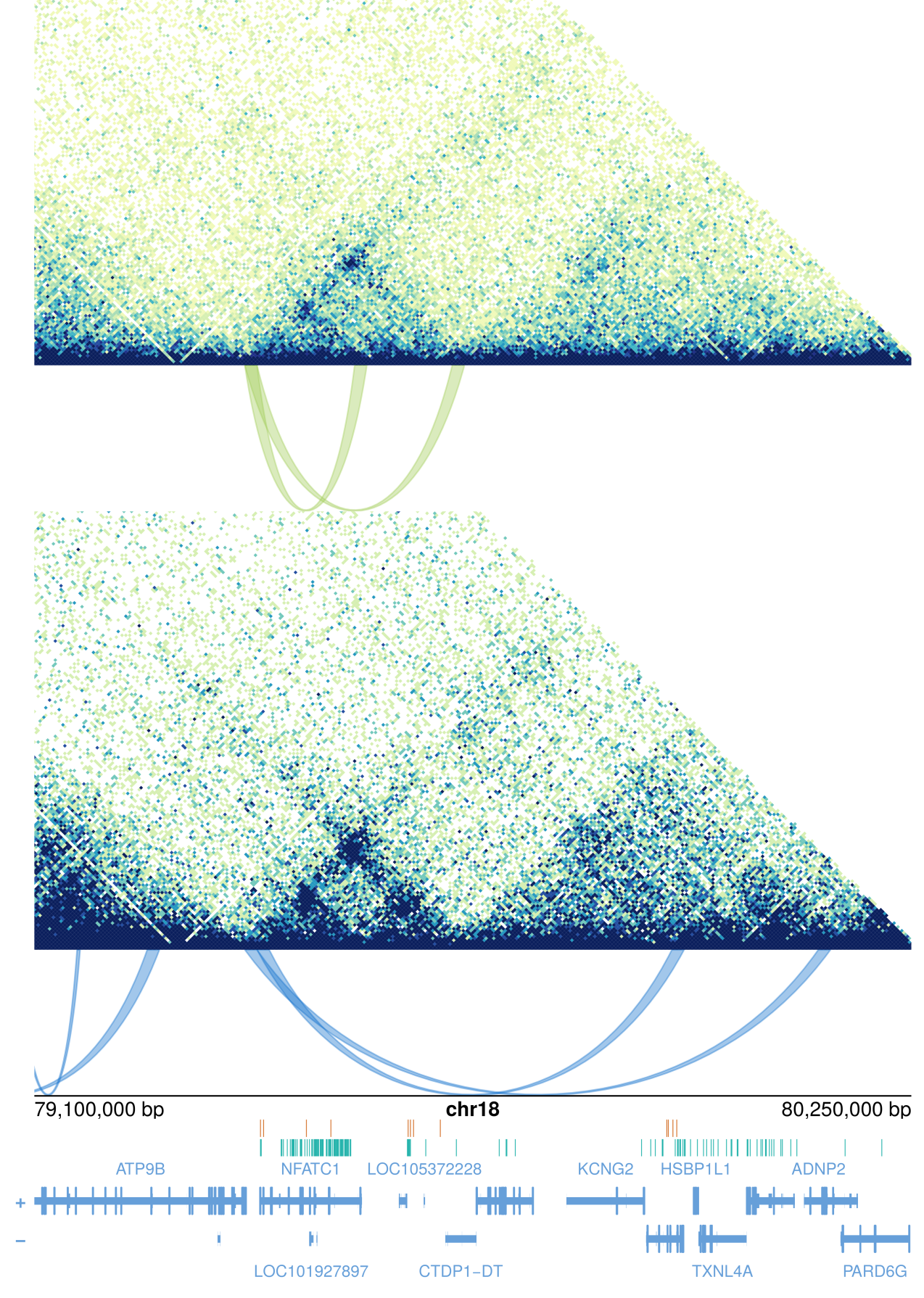


A.

B.

**Figure S5.** Micro-C data (topographical associated domains and chromatin interaction loops) in a region at chromosome 18q23, generated in A) human peripheral blood mononuclear cells (PBMC) and B) OC. Image shows the sentinel bone GWAS SNPs (**|**) and those in LD (r^2^>0.6) (**|**) and genes located on sense (**+**) and antisense strand (**–**) of the DNA. The upper right-hand portion of the contact matrix is truncated due to proximity to the telomere.


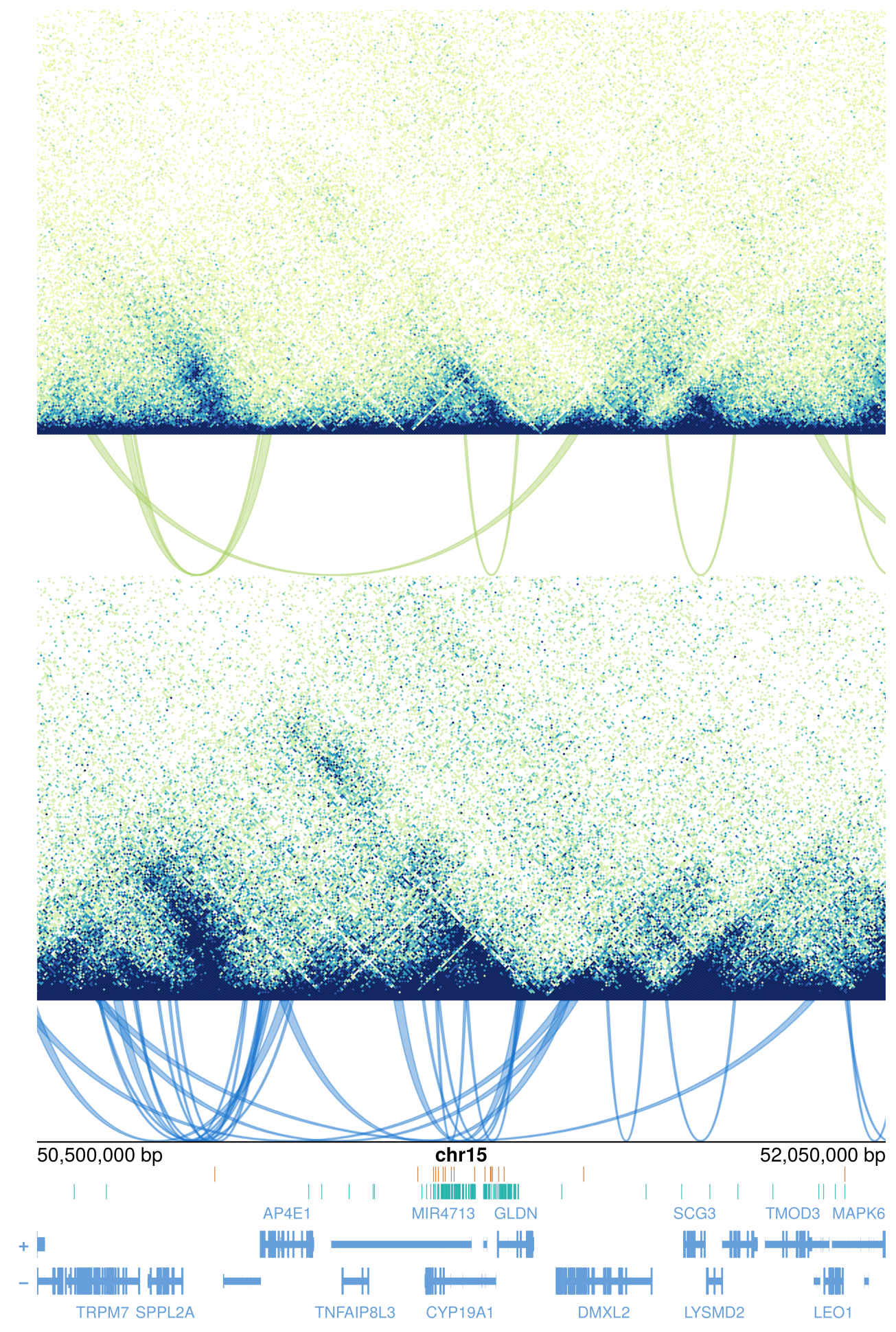


A.

B.

**Figure S6.** Micro-C data (topographical associated domains and chromatin interaction loops at 16kb resolution) at chromosome 15q21.2, generated in A) PBMC and B) osteoclasts (OC). Image shows the sentinel bone GWAS SNPs (**|**) and those in LD (r^2^>0.6) (**|**) and genes located on sense (**+**) and antisense strand (**–**) of the DNA.
